# A *Catalogus Immune Muris* of the mouse immune responses to diverse pathogens

**DOI:** 10.1038/s41419-021-04075-y

**Published:** 2021-08-17

**Authors:** Céline Barlier, Diego Barriales, Alexey Samosyuk, Sascha Jung, Srikanth Ravichandran, Yulia A. Medvedeva, Juan Anguita, Antonio del Sol

**Affiliations:** 1 Computational Biology Group, Luxembourg Centre for Systems Biomedicine (LCSB), University of Luxembourg, L-4362 Esch-sur-Alzette, Luxembourg; 2 Inflammation and Macrophage Plasticity laboratory, CIC bioGUNE-BRTA (Basque Research and Technology Alliance), Derio, 48160 Spain; 3 Department of Biological and Medical Physics, Moscow Institute of Physics and Technology, Dolgoprudny, Russian Federation; 4 Computational Biology Group, CIC bioGUNE-BRTA (Basque Research and Technology Alliance), Derio, 48160 Spain; 5 Institute of Bioengineering, Research Center of Biotechnology, Russian Academy of Science, Moscow, Russian Federation; 6 Department of Computational Biology, Vavilov Institute of General Genetics, Russian Academy of Science, Moscow, Russian Federation; 7 Ikerbasque, Basque Foundation for Science, Bilbao, Bizkaia 48012 Spain

**Keywords:** Immunology, Cell death and immune response

## Abstract

Immunomodulation strategies are crucial for several biomedical applications. However, the immune system is highly heterogeneous and its functional responses to infections remains elusive. Indeed, the characterization of immune response particularities to different pathogens is needed to identify immunomodulatory candidates. To address this issue, we compiled a comprehensive map of functional immune cell states of mouse in response to 12 pathogens. To create this atlas, we developed a single-cell-based computational method that partitions heterogeneous cell types into functionally distinct states and simultaneously identifies modules of functionally relevant genes characterizing them. We identified 295 functional states using 114 datasets of six immune cell types, creating a *Catalogus Immune Muris*. As a result, we found common as well as pathogen-specific functional states and experimentally characterized the function of an unknown macrophage cell state that modulates the response to *Salmonella* Typhimurium infection. Thus, we expect our *Catalogus Immune Muris* to be an important resource for studies aiming at discovering new immunomodulatory candidates.

## Introduction

The immune response to pathogens, such as viruses, bacteria, or fungi, is a complex process involving multiple immune and nonimmune cell types [1, 2]. Although transcriptional changes of these cells in response to pathogens have been studied for decades, the development of sensitive analytical techniques such as single-cell RNA sequencing (scRNAseq) only now enables the identification and functional characterization of cellular subpopulations in response to different stimuli. Thus, heterogeneous subpopulations can be identified by specialized transcriptional profiles that determine their identity and govern their interactions with invading pathogens [3–6]. Recent studies utilizing various pathogens have shown that complex transcriptional variability in macrophages govern their divergent response against individual invasive agents [7, 8]. For instance, in the case of *Salmonella enterica* Serovar Typhimurium, the interplay between the bacteria and macrophages triggers two different scenarios in which some cells are polarized to anti-inflammatory response whereas others display an inflammatory output [9]. Moreover, a subsequent study was able to identify two distinct cellular states that are responsible for a bimodal type I interferon response [10]. However, most of these studies focus on a single pathogen, making them unable to decipher common and distinct cellular states established in response to different infections. To date, only a few meta-analyses exist that aim at identifying common and unique patterns of the immune response to pathogens [11]. Nevertheless, these studies are based on the average response across a population of cells or tissues, making them unable to detect functionally distinct subpopulations. Moreover, the number of pathogens considered in these studies remains limited, which impedes more general conclusions regarding the cellular response to different types of infectious agents.

To date, several functional states of immune cells, such as macrophages, natural killer, and T cells, have been identified and characterized [12–15]. In general, discerning the functional states of immune cells and their transcriptional characterization is pivotal for the development of immunomodulatory therapeutic strategies. For instance, previous studies demonstrated the beneficial effect of reprogramming the macrophage polarization state to promote tumor suppression or alleviate autoimmunity in encephalomyelitis [16, 17]. However, the development of new immunomodulatory therapies based on the reprogramming of functional states is significantly impeded by the incomplete knowledge about the functional cell states established in response to pathogens and their characterization.

To address this challenge, we collected 114 single-cell datasets of six immune cell types in the context of 12 viral, bacterial, fungal, and parasite infections, and developed a computational method for identifying functional immune cell states in response to these pathogens, creating a *Catalogus Immune Muris*. We believe it will serve as a valuable resource of functional immune cell states to devise novel immunomodulatory strategies.

## Materials and methods

### Data collection, processing, and annotation

We collected 114 single-cell datasets composed of 6 immune cell types and 12 pathogens (Table S1). Raw data (accession numbers: PRJEB14043, E-MTAB-3857, and E-MTAB-4388) were processed using state-of-the-art pipelines [18]. Smart-seq data were subjected to a quality control step using fastqc, reads were mapped to the mm10 genome using STAR aligner and the count matrix were obtained using featureCounts tool. A similar workflow was applied for UMI-based data, adding the demultiplexing step and replacing the counting tool by umi-tool.

Datasets composed of several cell types were clustered using Seurat pipeline with default parameters, manually annotated and extracted. Cells were annotated using prior knowledge and CIPR web tool with default parameters [19]. Only the cells annotated with a good confidence were extracted and used to build the resource.

### Functional partitioning algorithm

In order to reliably identify and characterize functionally relevant cell states, we developed a network-based approach combined with a recursive hierarchical clustering named FunPart. The algorithm is composed of four main parts: (1) cleaning and normalization of the data, (2) network-based approach to identify set of genes strongly correlated, (3) functional characterization of the set of genes using manually annotated immune modules by Singhania et al. [11], and (4) recursive unsupervised hierarchical clustering to perform the splits. Each step is detailed in the Supplementary Information. A dataset for which no module is found is considered to be functionally homogenous and corresponds to one functional cell state.

### Validations and comparison with the state-of-the-art

We first aimed at validating our method at two levels: (1) the relevance of genes belonging to the detected functional modules, and (2) the relevance of the predicted cell states. We collected literature evidences for some of the main TFs identified in each module focusing on evidences of the immune process identified for macrophages. Next, we aimed at comparing our method with Seurat, a state-of-the-art method [20]. Seurat and FunPart were used with default parameters for the 17 macrophages datasets. We assessed the functional relevance of predicted clusters by both methods and computed a score reflecting the precision of each method in identifying real or artificial functional heterogeneity per dataset (Supplementary Information).

### Characterization of functional cell states

FunPart provides gene modules characterizing the predicted functional cell states as well as the specific immune process in which they are enriched. In order to have an additional layer of information, we aimed at identifying known markers to further characterize these cell states. Immune cell type markers were collected from the CellMarker database by considering experimentally validated evidences only [21]. We performed feature selection using the Boruta algorithm [22], a wrapper built around the random forest classification algorithm, to determine the importance of markers in classifying each cell states. Boruta was used in classification mode with default parameters for each cell state, details are provided in Supplementary Information. Fold changes and cell expression ratios were then computed for each cell states markers extracted by the algorithm (Supplementary Information).

### Metadata analysis

Data integration was performed for each dataset using the standard workflow of Seurat (Supplementary Information). Cell states were then aggregated across datasets for each cell type by following a hierarchical clustering approach: (1) Each dataset was first normalized individually by the third quantile to overcome the different types of expression values present in the different datasets (TPM, CPM, UMI and counts), (2) The median expression of each gene in each cell state was calculated, (3) Euclidean distance was then used to build the dendrogram reflecting the similarity between states, and (4) the dendrogram was splitted at a height corresponding to the seventh quantile of the heights distribution. The aggregated states were then embedded into the computed UMAP for visualization and analyses.

### Mice and bacteria

C57Bl/6 (B6) mice were purchased from Charles River Laboratories and bred in the Animal Facility at CIC bioGUNE. All the assays performed were approved by the competent authority (Diputación de Bizkaia) under European and Spanish directives. CIC bioGUNE is accredited by AAALAC Intl.

*Salmonella enterica* subsp. enterica serovar Typhimurium SL1344 (German Collection of Microorganisms and Cell Cultures, Leibniz, DE) was grown in Luria Bertani medium (Sigma–Aldrich) without antibiotics.

### Cell culture and gene silencing

Bone-marrow-derived macrophages (BMMs) were generated from 6–12-week-old B6 mice, as previously described [23]. Low-passaged HEK293FT cells were cultured in DMEM containing 10% FBS and 1% penicillin-streptomycin.

Lentiviral particles containing shRNA targeting *Zfp597* (TRCN0000215620, TRCN0000179758, TRCN0000245367, Sigma–Aldrich) and *Stat1* (TRCN0000235839) were generated using a third-generation lentivirus vector with a conditional packaging system [24, 25]. *Zfp597*-silencing in BMMs was conducted by co-infection with lentiviral particles containing the three silencing constructs whereas for *Stat1* one single construct was used. Lentiviral particles were added at days 3 and 5 of the differentiation process in the presence of 8 μg/ml protamine sulfate (Sigma–Aldrich). Controls were infected with lentiviral particles containing the empty vector, PLKO.1. BMMs derived from three independent mice were used in each silencing assay.

### Salmonella survival in murine macrophages

*S. typhimurium* was grown from a diluted (1:50) overnight inoculum until they reached an O.D. = 0.6. BMMs were infected following the protocol by Avraham et al. [10] at an m.o.i. of 10. In the experiments using shSTAT1 cells, 100 ng/ml of recombinant murine IFNγ was added at the same time than the bacteria. The mixture was centrifuged, incubated for 30 min, washed twice, and further incubated in the presence of 50 µg/ml gentamicin for 1 h. Macrophages were then washed and lysed in medium containing 0.1% Triton X-100. Cell lysates were centrifuged and resuspended in 1 ml of LB broth. Serial 1:10 dilutions were plated on LB-agar plates to determine the number of live intracellular bacteria per condition.

### Real-time PCR

Total RNA was isolated using the NucleoSpin® RNA kit (Macherey-Nagel) and reverse transcribed with M-MLV reverse transcriptase (Thermo Fisher Scientific). Real-time PCR was performed using the PerfeCTa SYBR Green SuperMix low ROX (Quantabio) on a ViiA 7^TM^ Real-Time PCR System (Thermo Fisher Scientific). Fold induction of *Zfp597* was calculated relative to *Rpl19* whereas *Stat1* was compared to *Actb* by using the 2^-ΔΔCt^ method. Standard curves of all primers were performed by testing serial dilutions of cDNA-experimental samples obtaining an average of 100 ± 5% efficiency. Correlation between target and housekeeping genes was assessed by standard curve comparisons (*Zfp597*-*Rpl19* slope 0.0194 / *Stat1*-Actin slope 0.0188). Details about the primers used can be found as Supplementary Information.

### Statistics

Three independent mice were used in each silencing assay. Data normality assumption was first validated using the Shapiro-Wilk test and variances between groups were analyzed using an F-test. Statistical difference between the two groups (control versus silenced assay) was then computed using a paired Student *t*-test. Results with a *p* value less than 0.05 were considered as being significant.

## Results

### Identification and characterization of functional immune cell states

**Fig. 1:**
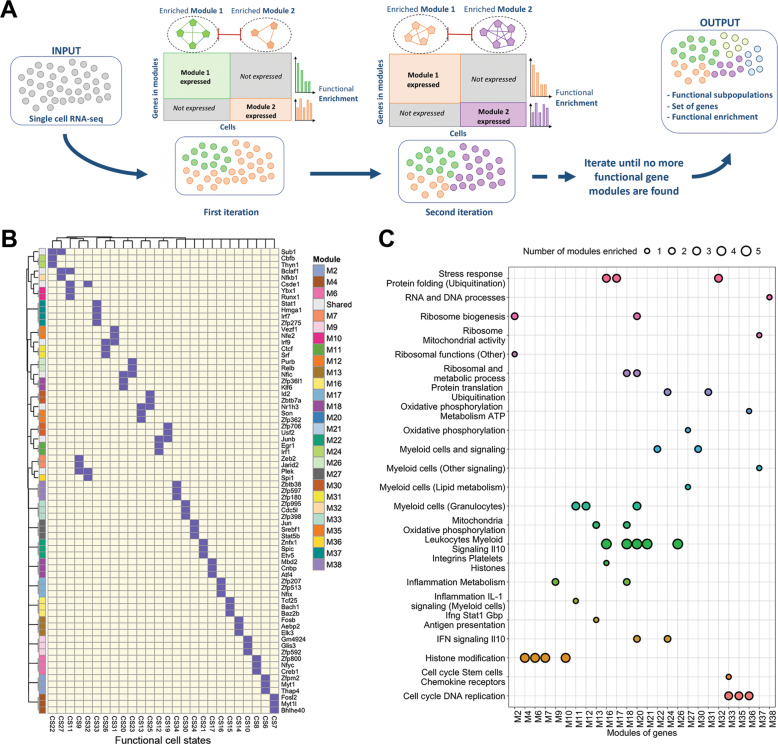
FunPart general workflow and validation. **A** General workflow of the functional states identification and characterization. The computational method we developed, named FunPart, takes single-cell RNA-seq data of one cell type as an input, to identify functional states based on functional modules of genes. The method searches for modules exclusively expressed in one group of cells and belonging to the same immune process. Cells are recursively splitted in two groups until no more functionally relevant modules associated to new states can be found. **B** Binary heatmap of the 26 terminal genes modules identified by FunPart for the macrophages functional cell states CS. Only TFs are displayed. **C** Functional enrichment of these 26 terminal gene modules. Each immune process has a different color, the size of the dots represents the number of gene modules enriched in the specific process. Intermediate gene modules are not displayed.

In order to create an atlas of functional immune cell states, we developed FunPart, a single-cell-based computational method that partitions heterogeneous cell types into functionally distinct states and simultaneously identifies modules of functionally relevant genes that characterize them. Starting from a population of cells belonging to the same cell type, the method partitions them into two subpopulations by searching for modules that are (i) exclusively expressed in one subpopulation and (ii) composed of co-expressed TFs belonging to the same immunological process. This procedure is recursively repeated until no functionally relevant modules, associated to new subpopulations, can be found (Fig. 1A).

To demonstrate the ability of this method to detect functional immune cell states, we collected 17 macrophage datasets corresponding to the infection with eight different pathogens profiled at different timepoints (Table S1). Application of our proposed method to these datasets revealed the presence of 9 M1-like, 13 M2-like cell states, and 14 middle range states expressing simultaneously some M1-like and M2-like markers [12] (Fig. S1). Moreover, literature evidences were found for every immune process and pathway reported by FunPart for the 12 intermediate genes modules, used to distinguish groups of functional states and 26 terminal gene modules, characterizing each individual state (Fig. 1B, C, Table S2). Next, we aimed at demonstrating that current clustering tools are unable to identify subtle functional differences and applied Seurat [20, 26], a widely used state-of-the-art clustering method, to each of the datasets. As expected, the subpopulations obtained are vastly different, with FunPart identifying 46% of functionally enriched ones compared to 33% for Seurat across the 17 datasets (Fig. S2A,B). Furthermore, FunPart distinguishes more accurately functional homogeneity and heterogeneity with 67% and 43% of true positives, respectively, compared to 25% and 22% for Seurat (Fig. S2C). In summary, FunPart identifies immune cell states more reliably and with an increased resolution compared to state-of-the-art methods.

### 295 functional immune cell states create a *Catalogus Immune Muris*

**Fig. 2:**
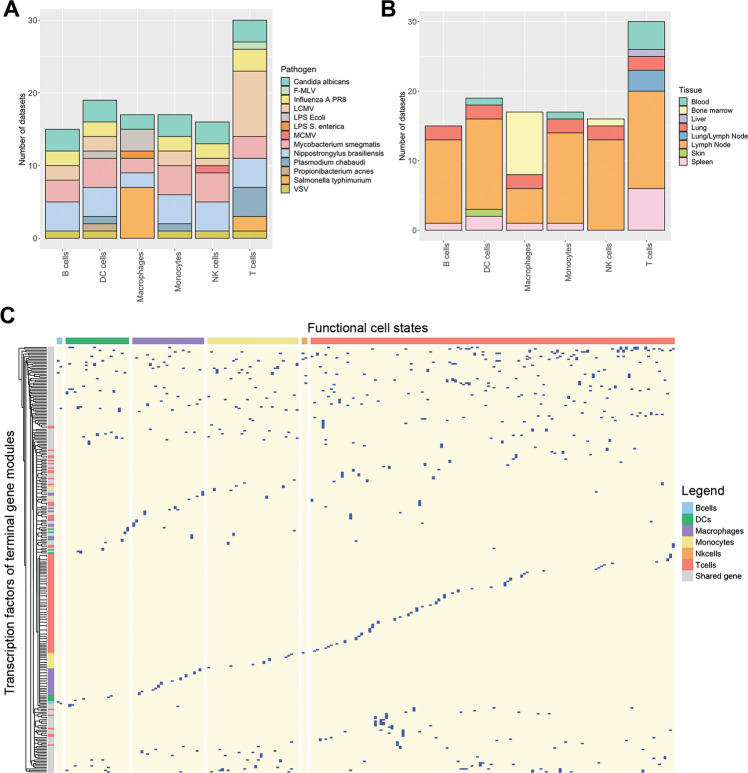
Overview of the *Catalogus Immune Muris* content. **A, B** Composition of the Catalogus Immune Muris. Repartition by immune cell type of the (**A**) 12 pathogens and (**B**) 8 tissues across the 114 datasets. LPS S. enterica and S. typhimurium are considered as the same pathogen. **C** Binary heatmap displaying the terminal gene modules identified by FunPart for each functional cell states belonging to one of the six broad immune cell type. Shared genes, colored in gray, correspond to transcription factors found in more than one terminal gene module. Only TFs of terminal gene modules are displayed.

**Fig. 3:**
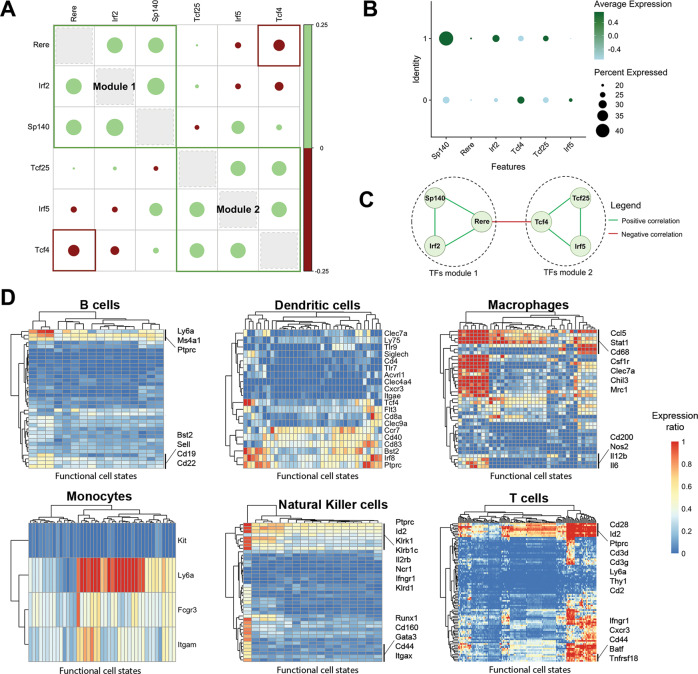
Functional cell states analysis and characterization. **A** Correlation plot and (**B**) dotplot of the functional TFs characterizing two B cells functional states in LCMV infection at timepoint 72 h. Colored boxes in (**A**) indicate correlations considered by the algorithm with green boxes indicating cliques of genes and red boxes the negative correlation considered as significant. **C** Network representation of the significant edges retained by the algorithm for the six TFs shown in (**A**). Each module consists of a clique of three transcription factors positively correlated together. The negative correlation between the two modules is supported by the interaction between *Tcf4* and *Rere*. **D** Heatmaps showing the expression ratio of the cell markers, extracted using Boruta, for each functional cell state. Identified cell states are in columns and markers in rows. A ratio of one corresponds to the marker being expressed in all cells of the functional cell state whereas a ratio of zero translates to the absence of its expression in the cell state.

After validating our approach for detecting functional cell states, we collected 114 single-cell RNA-seq datasets of B cells, T cells, natural killer (NK) cells, macrophages, monocytes, and dendritic cells (DCs) in the context of 12 viral, bacterial, fungal, and parasitic pathogens (Table S1). For each cell type we obtained data for six to nine pathogens across three to six tissues (Fig. 2A, B). Application of our method to these datasets resulted in the detection of 295 functional cell states in total, thus, creating a *Catalogus Immune Muris* (Fig. 2C, Table S3). On average, we identified 2.26 cell states per dataset and cell type, with NK cells and B cells having the lowest (average:1.06 and 1.07, respectively) and T cells having the highest (average: 4.45) functional heterogeneity. The low levels of functional heterogeneity in B cells are expected as their primary function is antibody secretion. Only in the context of lymphocytic choriomeningitis (LCMV), B cells exist in two distinct states characterized by two TFs modules composed of *Irf2*, *Rere*, *Sp140* for the first and *Irf5*, *Tcf25*, *Tcf4* for the second state, respectively (Fig. 3A, B, C). Moreover, *Irf5* is known to play a role in B cell differentiation [27] whereas *Irf2* is known to regulate B cell proliferation and antibody production [28], suggesting differences in the maturation stage of these cells. On the contrary, T cells exist in multiple cell states upon infection with various pathogens, such as LCMV, Influenza, and Salmonella Typhimurium. These are characterized by a marked difference in processes linked to stress response, inflammation and oxidative phosphorylation (Fig. S3). Interestingly, these processes are known to be involved in the functional diversity of T cells, more specifically by playing a role in their differentiation, activation, and function [29, 30]. Finally, we extracted known cell markers to further characterize the identified functional cell states (Fig. 3D, Table S4, S5). We found that combination of broad markers (e.g., CD3 for T cells) and specific markers (e.g., *Tlr9* for DCs) was important to classify the functional cell states, regardless of their relative expression (Table S4, Fig. S4). Finally, we further characterized functional states by identifying the expression of the extracted known cell markers for each functional state (Fig. 3D, Table S5). Interestingly, we observed few diversity in markers signatures for B and NK cell states whereas specific signature patterns were found for macrophages and T cells (Fig. 3D).

### Exploiting TF modules for modulating the inflammatory response

**Fig. 4:**
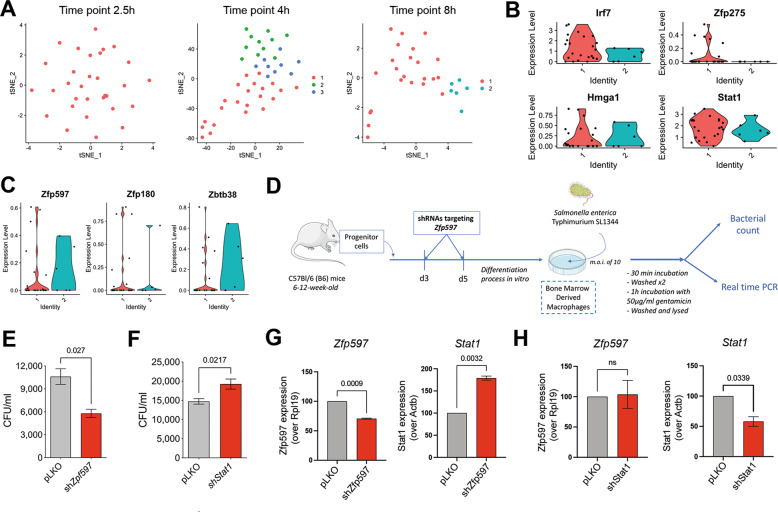
Immunomodulation of macrophage responses and functional states analysis. **A** t-SNE displaying functional states identified by FunPart across three timepoints for macrophages infected by Salmonella typhimurium. **B**, **C** Violin plots showing the expression levels for the two functional states identified at timepoint 8 h of (**B**) the first module composed of *Irf7*, *Zfp275*, *Hmga1*, *Stat1* and (**C**) the second module composed of *Zp597*, *Zfp180*, *Zbtb38*. **D** Summary of the experimental design used to validate *Zfp597* and *Stat1* as immunomodulators. **E–H** Differential survival of S. enterica typhimurium in *Zfp597*-silenced and *Stat1*-silenced macrophages compared to their respective pLKO controls. **E**, **F** Colony-forming units recovered from silenced and control-transfected BMMs infected with Salmonella at an m.o.i of 10 for (**E**) *Zfp597* and (**F**) *Stat1*. **G**, **H** *Zfp597* and *Stat1* gene expression levels in macrophages lentivirally infected with shRNAs targeting the gene or controls (plKO). The results are represented as average ± SE of three independent mice per silencing. The *p* values were calculated by paired Student’s *t*-test. A result is considered as significant if its *p* value is less than 0.05.

Due to the enrichment of TF modules distinguishing different cell states in immune cell processes, we hypothesized that the *Catalogus Immune Muris* can be exploited to modulate the inflammatory response to pathogens by perturbing the TFs characteristic of different states. In order to provide support to this hypothesis, we selected the macrophage response to *Salmonella enterica* Serovar Typhimurium [10] due to a characteristic temporal change in macrophage states during the infection. In particular, while only a single macrophage state can be detected 2.5 h after the infection, heterogeneity rapidly increases after 4 h (three states) and diminishes again after 8 h (two states) (Fig. 4A). By focusing on the two macrophage states detected 8 h after the infection, we found the first state to be characterized by the module containing *Irf7*, *Hmga1*, *Zfp275*, and *Stat1* (Fig. 4B) that has been previously shown to initiate the inflammatory response to pathogens in an interferon gamma dependent manner [10]. In contrast, the second state is characterized by a module composed of *Zfp597*, *Zbtb38*, and *Zfp180* (Fig. 4C), but lacks a functional characterization. Enrichment of these TFs and their co-expressed targets showed their involvement in RNA and DNA processes as well as pathways such as janus kinase (JNK) signal transduction (Table S2). Indeed, previous studies highlighted the importance of kinase activity in response to bacterial infection and the interference of pathogens with kinase-mediated phosphorylation as a beneficial strategy for bacterial survival, replication and dissemination [31, 32]. Thus, we hypothesized that macrophages exhibiting the second cell state are not responding to *Salmonella* infection due to kinase-mediated phosphorylation of proviral signaling pathways. We sought to validate this hypothesis by knockdown of *Zfp597* as this TF had the strongest co-expression pattern with its targets in the cell state characterized by the gene module. Therefore, we assessed the survival of *Salmonella* in primary murine bone-marrow-derived macrophages after silencing *Zpf597* with shRNA lentiviral constructs during the differentiation process [23] (Fig. 4D). The results in three independent mice showed that silencing of *Zpf597* resulted in a decreased ability to recover viable bacteria upon 90 min incubation periods demonstrating that *Zpf597* is responsible for preventing the macrophage response to *Salmonella* infection (Fig. 4E). Thus, the subpopulation characterized by the module involving *Zfp597* is indeed not responding to the pathogen due to the propathogenic effects of *Zfp597* and its inhibition induced a change in cell state. To further support the induced macrophage state change, we employed the same experimental setup to silence *Stat1* and hypothesized that bacterial survival is increased. Indeed, recovery of viable bacteria upon 90 min incubation periods in the presence of IFNγ demonstrated that *Stat1* is a driver of bacterial clearance (Fig. 4F), which is consistent with previous reports [33, 34]. Moreover, we analyzed the expression of both silenced TFs on their respective TF module counterparts in order to determine regulatory relationship between the two modules (Fig. 4G, H). We observed that silencing of *Zfp597* induced a significant increase in *Stat1* expression whereas *Stat1* silencing did not significantly alter *Zfp597* expression (Fig. 4G, H). This suggests a regulatory relationship between the two modules, with *Zfp597* inhibiting the expression of *Stat1*, which belongs to the opposite module.

In summary, the TFs characteristic of the detected cell states could be harnessed to modulate the immune response to pathogens by inducing a transition of cell states.

### Integration across pathogens identifies common and unique cell states in time and space

**Fig. 5:**
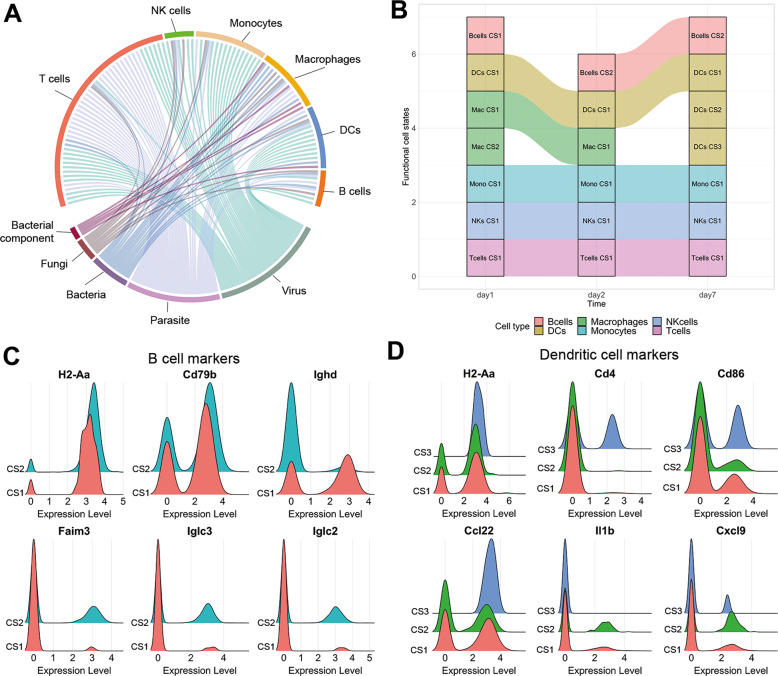
Metadata analysis of functional cell states. **A** Chord diagram representing the common and unique cell states across pathogen types infections. **B** Alluvial plot of the functional states identified for the six immune cell types infected by Mycobacterium smegmatis across three timepoints. CS functional Cell State, DCs dendritic cells, Mac macrophages, Mono monocytes, NKs Natural Killer cells. **C**, **D** Markers expression for (**C**) the two B cells and (**D**) the three DCs functional cell states shown in (**B**). Distributions displayed include all applicable timepoints.

As previously described, a major bottleneck of previous studies is the inability to compare the immune response across pathogens and timepoints. To address this issue, we set out to unify the previously detected cell states across different datasets by combining similar states. As a result, we obtained between 5 and 45 unique states for each cell type. We observed that the majority of functional states is homogeneous although some states display heterogeneous functionalities shared by other states (Fig. S5). Similar to the analysis conducted for individual datasets, NK and B cells have the lowest number of unique states whereas T cells have the highest. Next, we leveraged this integrated collection to identify functional states common and unique in the response to different pathogens. Interestingly, we observed largely distinct responses to different types of pathogens for most of the cell types, underscoring the previously reported predominance of pathogen-specific immune responses (Fig. 5A) [35]. Finally, we set out to interrogate the changes in cell states at different timepoints of an infection. We analyzed the *Mycobacterium smegmatis* infection for the six cell types and observed a conserved functional state for T cells, NK cells, and monocytes across the three timepoints, respectively (Fig. 5B). Indeed, no functional diversity is observed for T cells, which are in one conserved state across the 7 days. However, B cells and DCs have conserved and unique states, with the functional diversity of DCs increasing at day 7. We noticed a shift of functional B cell states between the first and second day, mainly characterized by the differential expression of IgD (Fig. 5C) [36]. Furthermore, we observed that the functional diversity of DCs at day 7 is characterized by three functional states (Fig. 5D) and could reflect differential DCs maturation during the inflammatory response, as reported in previous reports [37]. In addition, the functional state CS3 is the most different with the expression of *Cd86*, *Cd4*, and especially *Ccl22*, suggesting this state to be actively recruiting other cells, such as invariant NKT or regulatory T cells, in response to the infection [38–41].

## Discussion

In this study, we developed FunPart, a single-cell-based computational method to dissect the heterogeneous cellular response of immune cells to pathogens. In particular, this method is conceptually different from traditional clustering methods [42] as it accounts for functional aspects by identifying specific set of genes required to belong to the same immune process. Moreover, the striking difference between our approach and current clustering methodologies can be exemplified in the context of B cell states. Although traditional clustering methods detected 11 memory B cell states in a recent study, only a few states exhibited significant differences [43]. This is in accordance with our observation that B cells do not exhibit a high functional diversity with respect to immune processes. Furthermore, it was not unexpected to identify the largest number of functional states for T cells [44, 45]. The differential diversity between B and T cells was observed at the marker expression level, initially used to distinguish cells (sub)types [21], but not fully explanatory of the functional diversity captured. Thus, the main advantage of our approach is that it mainly captures functional rather than transcriptional heterogeneity. Moreover, FunPart provides modules of genes used to identify the functional cell states and the immune processes [11] to which they belong. As a result, we were able to compile a *Catalogus Immune Muris*, the most comprehensive atlas of immune cell states currently available to the research community.

In addition, the *Catalogus Immune Muris* contains a molecular characterization of each state that can be leveraged to design novel immunomodulatory strategies. Here, we showed that the cellular response to *Salmonella* infection can be modulated by inhibiting TFs from identified gene modules by FunPart to enhance or inhibit pathogen clearance. Indeed, as reported in previous studies, we found *Stat1* to be a driver of bacterial clearance [33, 34], whereas we identified *Zfp597*, a previously unreported TF, to have propathogenic effects. We showed that perturbation of TFs predicted to be characteristic of two macrophage cell states allows the modulation of their response to the infection by a switch between functional cell states. Moreover, our analysis suggests a regulatory relationship between the two modules where *Zfp597* inhibits the expression of *Stat1*. Therefore, targeting the identified TFs provides a rationale strategy for immunomodulatory therapies [46, 47]. Nevertheless, the development of novel immunomodulatory therapies typically relies on the utilization of drugs and compounds to alter cellular functions [48, 49]. In this regard, a limitation of the presented strategy is that it solely considers modules composed of transcription factors that are potentially difficult to target.

Finally, the strategy implemented in FunPart could be of use for deciphering and characterizing functional heterogeneity within cell populations in diverse pathological and physiological conditions. Indeed, our method is not biased by the cell type it analyzes and thus could be applied to any cell type in any tissue or condition. Although FunPart currently identifies modules enriched in immune cell processes, it can be easily adapted to other genesets characteristic of any biological process. For instance, it could be applied to study the functional impairement of cell (sub)types in liver-related diseases [50, 51]. Indeed, it is known that the cellular location around the lobule plays an important role for their function [52], however the dysregulations imparing the hepatocytes functions is not well defined [51, 53]. The identification and characterization of such functional subtypes could help improving regenerative medicine strategies [54].

In summary, we presented a computational strategy for resolving functional cell states in the context of infections and identifying TFs involved in the maintenance of these states. We expect our approach to be of great utility for deciphering and characterizing functionally distinct cell states in physiological and pathological conditions. Moreover, application of our method to 114 datasets created a *Catalogus Immune Muris*, which we believe to be of great utility in the development of novel immunomodulatory therapies.

## Supplementary information

Supplementary Information

Supplementary Table S1

Supplementary Table S2

Supplementary Table S3

Supplementary Table S4

Supplementary Table S5

## Data Availability

The accession number of the datasets used are available in the table S1. The integrated datasets for each cell type are available at: https://gitlab.com/C.Barlier/immunofunmap.git. The maps are available via an interface developed with Shiny at: https://gitlab.com/C.Barlier/immunofunmap.git.

## Code availability

The method implemented to identify and characterize the functional cell states is an R package named FunPart available at: https://github.com/BarlierC/FunPart.git.
