# Supplementary material for: A Catalogus Immune Muris of the mouse immune responses to diverse pathogens
Source: Cell Death Dis. 2021 Aug 17;12(9):798. doi: 10.1038/s41419-021-04075-y (PMC8370971; doi:10.1038/s41419-021-04075-y)
Supplement: Supplementary file 1 — Supplementary Information [file 41419_2021_4075_MOESM1_ESM.docx]

**Supplementary Information**

# **Table of content:**

**Supplementary Methods**

**Fig. S1.** M1-like and M2-like markers used to classify macrophages functional states.

**Fig. S2.** FunPart validation and comparison to state-of-the-art.

**Fig. S3.** Enrichment of the terminal and intermediate gene modules identified for T cells.

**Fig. S4.** Extracted features for each functional cell state.

**Fig. S5.** UMAPs of the integrated data for the six immune cell types.

**Table S1.** Single cell datasets used to build the resource.

**Table S2.** Validation of the functional modules predicted by FunPart.

**Table S3.** *Catalogus Immune Muris* functional cell states and molecular characterization.

**Table S4.** Extracted features for the functional cell states using Boruta.

**Table S5.** Cell markers signatures for each functional cell state.

# **Supplementary Methods**

# **Functional partitioning algorithm**

In order to reliably identify and characterize functionally relevant cell states, we developed a network-based approach combined with a recursive hierarchical clustering named FunPart. The algorithm is composed of four main parts:

1. Cleaning and normalization of the data: The algorithm accepts any type of data (counts, UMI or normalized). In case raw data are provided, a normalization procedure will be performed using Seurat. Outliers cells will be removed using a Rosner test on the number of genes expressed in each cell. Finally, two last quality control steps are performed on the genes: (1) any gene expressed in less than 5% of the cells will not be considered and (2) genes that are too lowly expressed are removed, with a gene too lowly expressed falling below the 5% of genes expression sum distribution.
2. Network-based set of genes identification: To identify functional sets of genes, a correlation network is constructed around all the genes. Based on the correlation scores distribution, the 2.5% of each tail are considered to be the strongest interactions and are kept for the following steps: (1) identification of cliques of transcription factors (TFs) that are positively correlated together, (2) filtering of cliques that are not unique, with a unique clique defined as a clique with less than 70% of common TFs, (3) an expression score reflecting the average expression of the clique is calculated and only the top 30% is kept, (4) in order to identify antagonistic pairs of cliques, a negative score is calculated between each pair of positive clique identified in 3., (5) if less than ten antagonistic pairs are found, all of them are used in step 6., however if more than ten are found, only the top 5% most negatives are kept, (6) the top target genes are identified for each TF of the two modules.
3. Functional characterization of the set of genes: An enrichment analysis is then performed on the candidate pairs of modules, using manually annotated immune modules by Singhania et al. The functional enrichment is performed using the clusterProfiler R package as the following: (1) all the genes profiled in the dataset are used as the universe and genes of the module considered are used to perform the comparison, (2) a multiple test correction (Bonferroni) is performed and only the enriched annotations with an adjusted p-value less than 5% are kept, (3) enriched categories mapped to only one gene of the set are not considered, (4) a score consisting in the sum of all the resulting gene ratio for the module is computed, (5) the two negatively connected modules need to be both enriched to be considered, (6) each pair of negatively connected modules is ranked according to the computed score. The top one enriched set, consisting of two gene modules, is then used for the hierarchical clustering.
4. Recursive unsupervised hierarchical clustering: In order to investigate each level of resolution, a recursive binary splitting is used (unsupervised hierarchical clustering). For each level, a bi-clustering is performed by building a heatmap using the cells of the corresponding level as well as the identified genes of the two gene modules. The general workflow is the following: (1) at each level, a hierarchical tree is constructed using the single cell expression data, the best set of genes and the Pearson correlation measure using the complete aggregation approach, (2) the first level of the cells dendrogram is used to perform the binary cutting with k = 2, (3) the two distinct groups of cells identified will then be splitted separately as explained in steps 1 to 2. The algorithm stops once the groups of cells are homogeneous and no more functional gene modules are found.

FunPart deciphers functional diversity by identifying and using set of gene modules to pinpoint and characterize functional cell states. Each gene module identified is composed of TFs, forming a clique of positively co-expressed edges only, and their direct neighbor genes for which they have a strong positive interaction. Furthermore, these genes modules can be classified as intermediate modules or terminal modules. A genes module is intermediate if the group of cells identified is further splitted whereas a genes module is terminal if the group of cells identified is not further splitted (corresponds to a functional cell state and leaf in the hierarchical tree). Indeed, an intermediate gene module characterize a group of functional cell states whereas a terminal gene module characterizes a specific functional cell state.

The module attribution to a group of functional cell states or one functional cell state is performed for each binary splitting. Indeed, each binary splitting is performed using two gene modules, with each of them belonging to one of the two groups resulting from the split, according to FunPart rationale. Thus, the module attribution is performed based on the average number of cells expressing the TFs of the clique in the module. Each gene module is then assigned to the group (branch 0 or 1) in which it is expressed the most and classified as characterizing this group. This step allows the assignment of intermediate gene modules to group of functional cell states and terminal gene modules to specific functional cell states.

**Validations and comparison with the state-of-the-art**

The functional relevance of the predicted subpopulations by FunPart and Seurat was assessed as follow: for each dataset, a ROC test, using FindAllMarkers function from Seurat R package, has been applied to each predicted cluster; genes with an AUC greater or equal to 0.7 were considered as good candidates to classify the group of cells; genes were submitted to an enrichment analysis using annotated immune modules, a Benjamini-Hochberg correction and a p-adjusted value less than 5%. We then defined four classes to assess the functional relevance of the predictions based on each dataset:

- “True homogeneous”: dataset for which one method do not identify subpopulations and the other one identifies some from which more than 50% are non-functional;
- “False homogeneous”: dataset for which one method do not identify subpopulations but the other one identifies some from which more than 50% are functionally relevant;
- “True heterogeneous”: dataset for which more than 50% of the cell states identified are functionally relevant;
- “False heterogeneous”: dataset for which less than 50% of the cell states identified are functionally relevant.

The four non splitted datasets by both methods were discarded from this analysis. We computed a precision score such as precision = True / (True + False).

**Characterization of functional cell states**

The feature extractions were done using the R version of Boruta’s algorithm, a wrapper built around the random forest classification algorithm, for each functional cell state. Boruta was used with default parameters and the following predictors and response vector:

- Predictors: matrix with features in columns and cells in rows. The features used consisted of the collected markers for the broad cell type of the functional cell state.
- Response vector: vector with two classes (binary classification), with class 1 for the cell state under consideration and class 0 for all the other cell states (background).

For each functional cell state, we kept markers classified as an important feature and then computed a fold change (FC) such as:

$$FC=\frac{mean(x_{m}^{cs})}{mean(x_{m}^{b})}$$

*With m: marker, cs: functional cell state, b: background, x: gene expression.*

A positive FC represents an overexpression of the marker in the functional cell states whereas a negative one represents a down-expression of the marker.

In order to compile markers profile for each functional cell states we identified, we computed cell expression ratios for each functional cell state and each extracted feature of the immune cell types. The ratios were computed for each functional cell states such as:

$$R_{cs}^{m}= \frac{\sum x_{i}^{m}}{n_{cs}}$$

*With* $R$*the ratio, m the marker, cs the functional cell state, x the binary expression (0 or 1, with 1 = expressed) of the marker m in the cell i and n the total number of cells.*

**Metadata analysis**

Data were integrated using the standard Seurat pipeline. Due to the high disparity between the number of cells, the integrations were performed in three steps with the biggest datasets (>1000 cells) being integrated together and then, integrated with the medium ones (>100 and <1000 cells) to finally be integrated with the smallest ones. The UMAP is computed, for each cell type, on the integrated data using Seurat and the functional set of genes characterizing the functional cell states identified using the functional splitting algorithm.

**Real time PCR**

The primers used corresponded to the genes Rpl19 (5’-GAC CAA GGA AGC ACG AAA GC-3’ and 5’-CAG GCC GCT ATG TAC AGA CA-3’), Zfp597 (5’-ATC GGA TGA GCA GAG ACC AC-3’ and 5’-TGA ACA ACG GGT GCA GCA AT-3’), Stat1 (5’ -TCT GAA TAT TTC CCT CCT GGG- 3’ and 5’ -CGG AAA AGC AAG CGT AAT CT- 3’) and Actb (5´-GAC GAT GCT CCC CGG GCT GTA TTC-3´ and 5´-TCT CTT GCT CTG GGC CTC GTC ACC-3´).

**
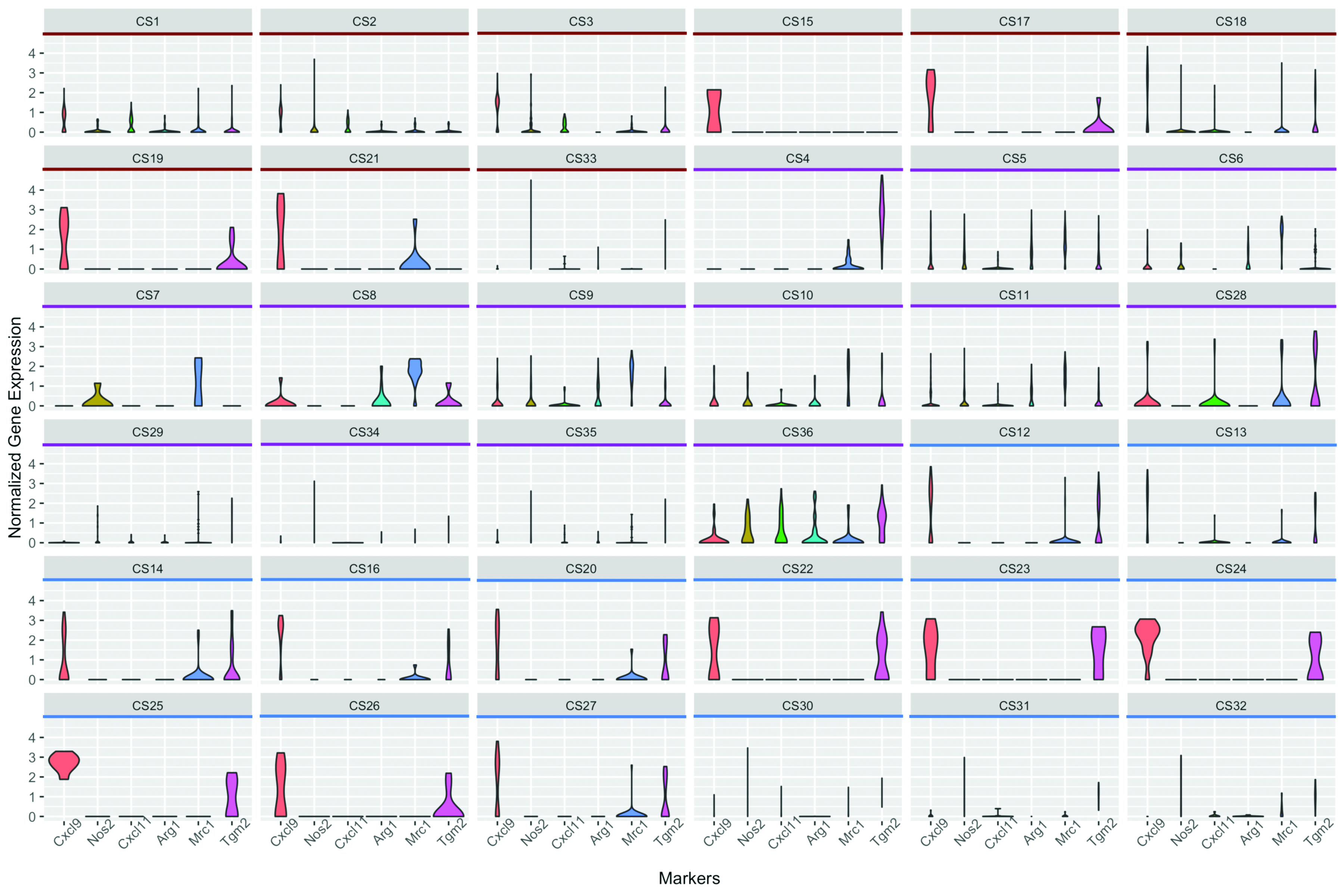
**

**Fig. S1.** **M1-like and M2-like markers used to classify macrophages functional states.** Representation of the M1-like (Cclx9, Nos2, Cxcl11) and M2-like (Arg1, Mrc1, Tgm2) markers distribution used to classify macrophages functional cell states (CS) as M1-like, M2-like and intermediate. CS underlined in red are classified as M1-like, CS underlined in purple are classified as M2-like and CS underlined in blue are classified as intermediate states.

**
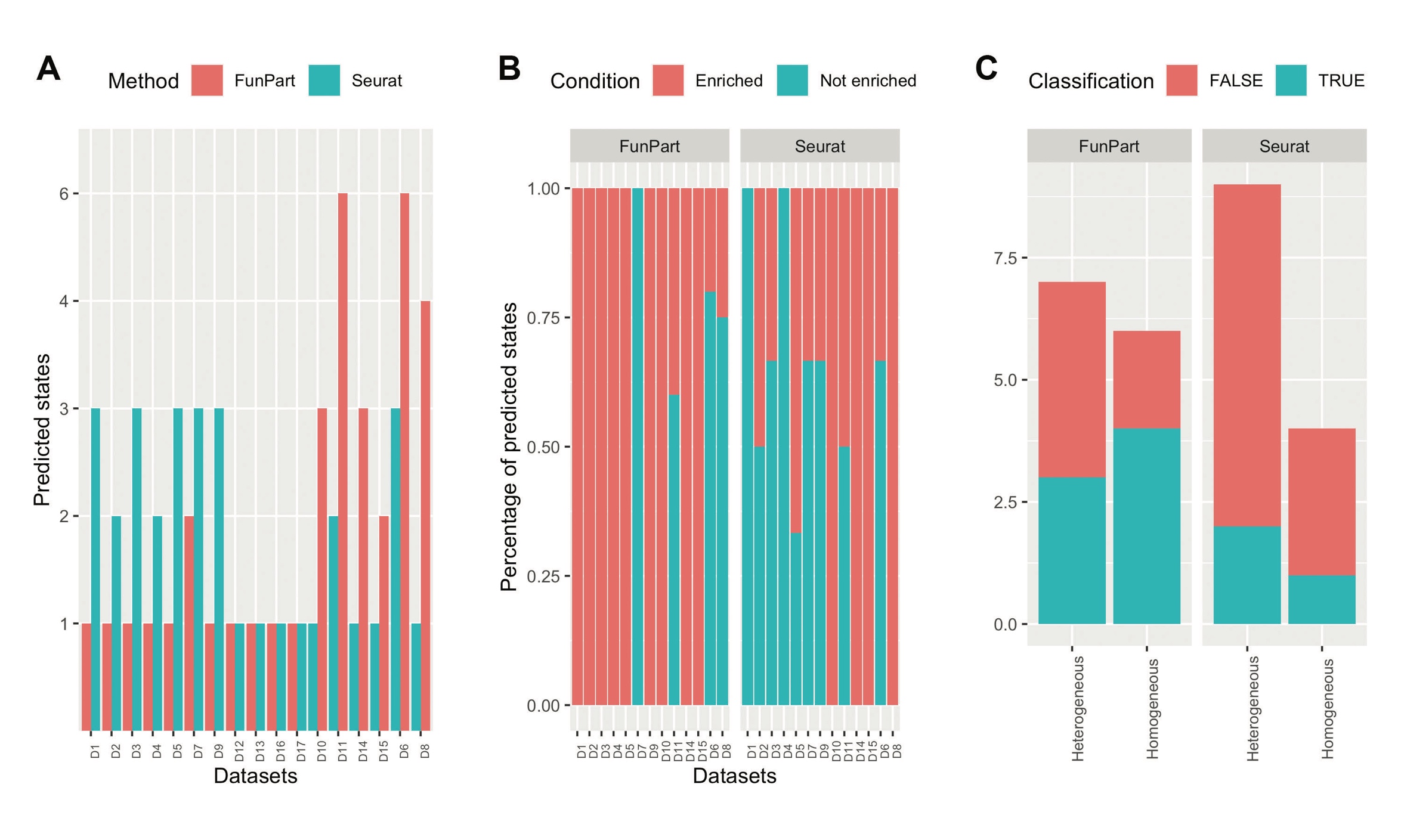
**

**Fig. S2. FunPart validation and comparison to state-of-the-art. (A)** Predicted states by FunPart and Seurat for the 17 macrophages datasets. **(B)** Ratio of enriched and non-enriched predicted subpopulations for the 14 datasets for which FunPart and Seurat were not in agreement. Datasets D12, D13, D16 and D17 have been excluded from this analysis. **(C)** Assessment of the accuracy of both methods in distinguishing functional homogeneous datasets and identify functionally relevant subpopulations (True Heterogeneous). The computation of the different classifications is described in the Methods section.


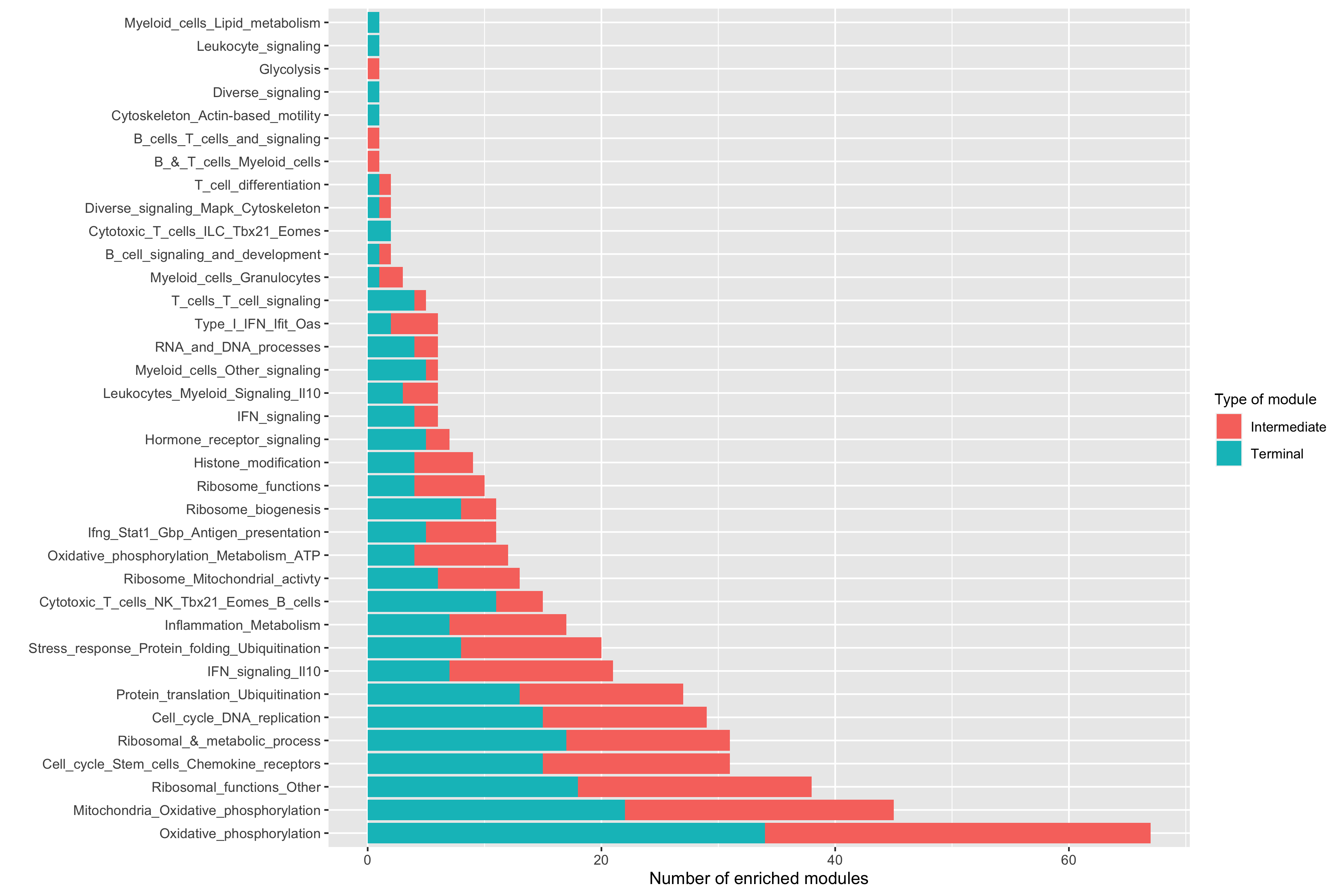


**Fig. S3. Enrichment of the terminal and intermediate gene modules identified for T cells.** FunPart identified 132 terminal and 102 intermediate gene modules across the 30 T cells datasets analyzed that were enriched in diverse immune processes. Most of the modules are enriched in processes involved in broad processes such as oxidative phosphorylation, stress response and inflammation metabolism whereas fewer are enriched in more specific ones such as type I IFN and cytotoxic T cells processes.

**
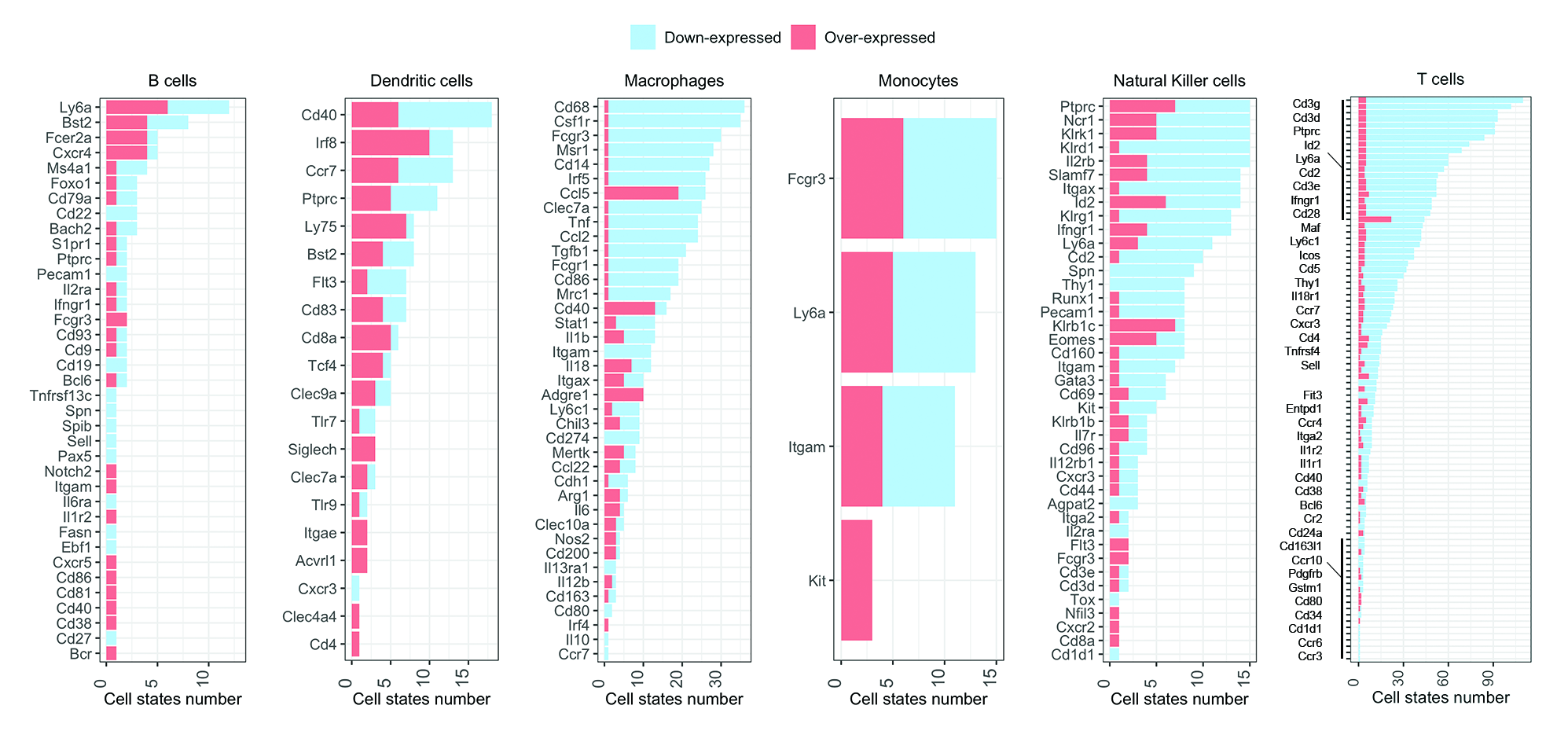
**

**Fig. S4. Extracted features for each functional cell state.** Feature extraction was performed to identify important marker to classify the identified functional cell states. Stacked boxplots represent the frequency of each marker being found as important for the classification. Light blue parts represent markers found down-expressed in the specific functional cell considered and red parts represents over-expressed markers. We can observe that broad markers such as CD3 for T cells are more frequent than specific markers such as Tlr9 for dendritic cells, regardless of their expression level.


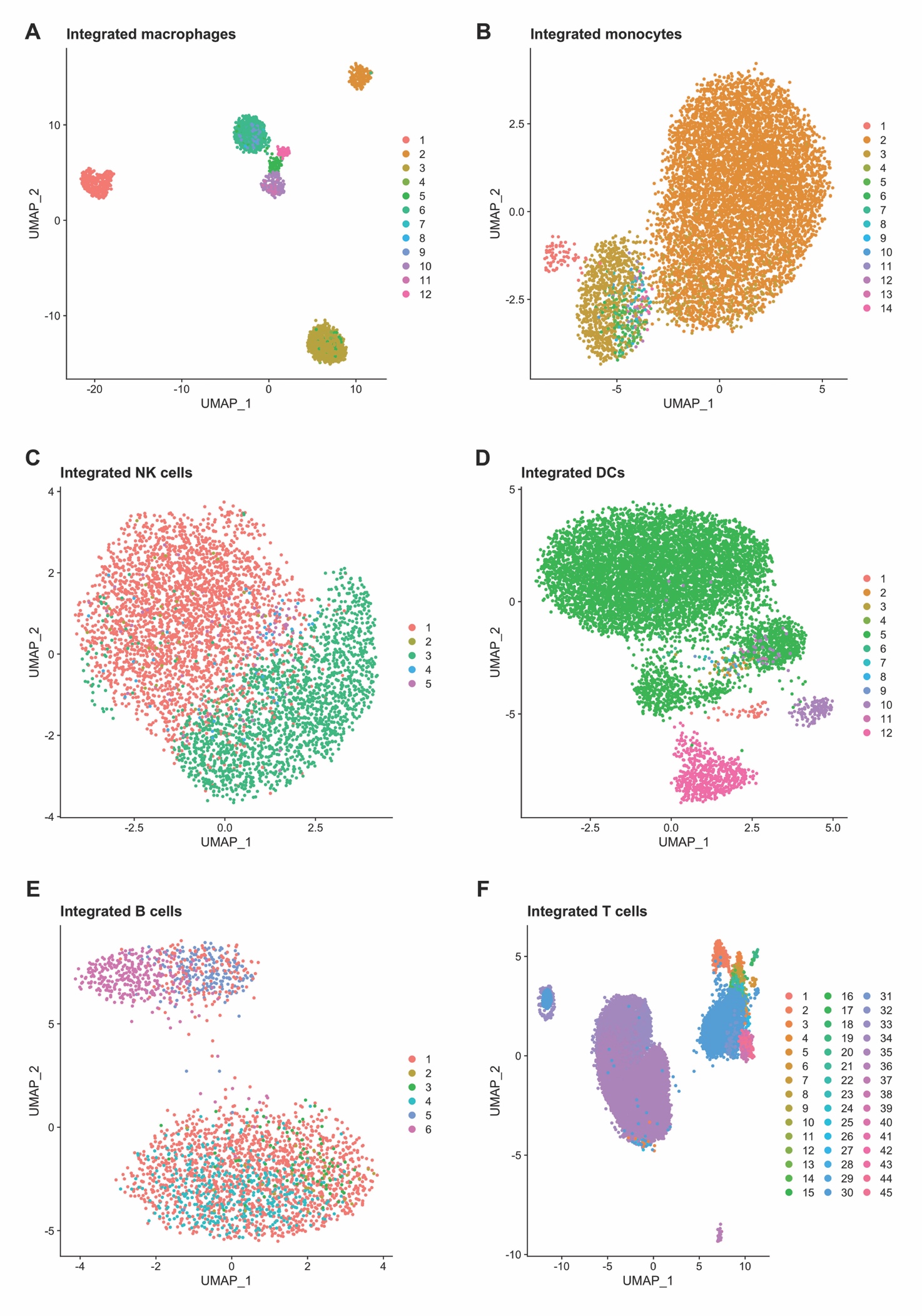


**Fig. S5. UMAPs of the integrated data for the six immune cell types.** UMAP were computed for integrated data of the **(A)** macrophages, **(B)** monocytes, **(C)** natural killer (NK) cells, **(D)** Dendritic cells (DCs), **(E)** B cells and **(F)** T cells. They were built using all functionally relevant genes reported by FunPart for the non-integrated analysis for each cell type as features. Functional states identified after the integration analysis are displayed with some of them in intermediate states and not distinct. NK cells and B cells have the lowest number with 5 and 6 respectively compared to the T cells composed of 45 functional states.
